# Vaccine uptake, barriers and enhancers of COVID-19 vaccination among healthcare workers from high-burden cities in Ethiopia

**DOI:** 10.4102/jphia.v16i1.673

**Published:** 2025-04-09

**Authors:** Gutema B. Tura, Derbachew A. Teni, Saro Abdella, Jaleta B. Tura, Yakob Wondarad, Getahun Fetensa, Tesfaye Gelanew, Alemseged Abdissa, Senga Sembuche, Elizabeth Gonese, Tamrat Shaweno, Nebiyu Dereje, Mosoka P. Fallah, Leah Mbabazi, Rodgers R. Ayebare, Agnes Kiragga, Francis Kakooza, Mesay Hailu, Getachew Tollera, Raji Tajudeen, Aster Tsegaye

**Affiliations:** 1 Ethiopian Public Health Institute, Addis Ababa, Ethiopia; 2 Ministry of Health, Addis Ababa, Ethiopia; 3 Armauer Hansen Research Institute, Addis Ababa, Ethiopia; 4 Africa Centres for Disease Control and Prevention, Addis Ababa, Ethiopia; 5 Infectious Diseases Institute, Makerere University, Makerere, Uganda; 6 Department of Medical Laboratory Sciences, College of Health Sciences, Addis Ababa University, Addis Ababa, Ethiopia; 7 Ethiopian Public Health Association, Addis Ababa, Ethiopia; 8 African Forum for Research and Education in Health Limited by Guarantee, Kumasi, Ghana

**Keywords:** COVID-19, COVID-19 vaccination, vaccine uptake, healthcare workers, Ethiopia

## Abstract

**Background:**

Coronavirus disease 2019 (COVID-19) vaccination is crucial for healthcare workers (HCWs) to protect themselves and promote public health.

**Aim:**

This study assessed COVID-19 vaccination uptake, barriers and enhancers among HCWs in high-burden cities in Ethiopia.

**Setting:**

A cross-sectional survey among 600 randomly selected HCWs from 30 health facilities (May 2023 to July 2023).

**Methods:**

Interviewer-administered questionnaires assessed vaccination status, concerns and motivators. Descriptive statistics and modified Poisson regression identified factors associated with vaccination.

**Results:**

Overall, 70.3% (*n* = 422) received at least one dose, and 39.2% (*n* = 235) were fully vaccinated. Safety concerns (51.9%) were the main barrier. More information on safety and efficacy (53.1%) and regulatory approval (27.3%) were key motivators. Those not recommending vaccination were less likely to be vaccinated (prevalence ratio [PR] = 0.59, 95% confidence interval [CI]: 0.41–0.85). Professionals such as radiographers (PR = 0.78), pharmacists (PR = 0.79) and laboratory personnel (PR = 0.85) were less likely compared to physicians. Older HCWs (> 25 years) were twice as likely to be vaccinated. HCWs in health centres were more likely to be vaccinated than those in hospitals (PR = 1.201, 95% CI: 1.076–1.341).

**Conclusion:**

A significant proportion of HCWs were not fully vaccinated. Targeting hospital workers and younger age groups and improving HCWs confidence in recommending vaccination can increase uptake.

**Contribution:**

This study reveals COVID-19 vaccine safety, efficacy and confidence concerns of HCWs, highlighting the need for targeted awareness to strengthen national vaccination efforts against pandemics.

## Introduction

The coronavirus disease 2019 (COVID-19) pandemic, which is caused by severe acute respiratory syndrome coronavirus 2 (SARS-CoV-2), has been a global public health emergency.^1^ The world experienced different waves of the pandemic with varying magnitude. As of 13 December 2023, there have been 772 386 069 confirmed cases of COVID-19 reported to World Health Organization (WHO), including 6 987 222 deaths. In Africa, there were 9561 535 confirmed cases and 175 418 deaths. During the same period, 501 117 confirmed cases and 7574 deaths were reported in Ethiopia.^2^

The COVID-19 vaccines have effectively reduced transmission, morbidity and mortality.^3^

Mass vaccination is a proven strategy for protecting susceptible individuals in the population.^4^ Africa Centers for Disease Control and Prevention (CDC) partnered with the Mastercard Foundation through the Saving Lives and Livelihoods Program (SLL) to vaccinate 70% of Africa’s 3.3 billion population, as per the WHO global vaccination target.^5^ However, only 34% of the African population was fully vaccinated until 20 July 2022, and Mauritius was the only African Union member state that has achieved this WHO global vaccination target.^2^

As of 26 November 2023, 13.59 billion vaccine doses have been administered globally.^2^ In Ethiopia, 68 856 793 vaccine doses have been administered as of 24 December 2023.^2^ At the start of vaccination campaigns in Africa because of the scarcity of vaccines, priority was given to health workers, teachers and older people. However, the need for global vaccine equity has increased the supply of vaccines across Africa. Despite the increased access to efficacious COVID-19 vaccines, many remain unvaccinated because of prevailing misconceptions about the vaccines. As a result, only 14.14% of the eligible population are partially vaccinated, 19.5% were fully vaccinated, and only 0.35% had received booster doses.^6^ Cognisant of this, the Africa CDC aimed to support countries in understanding vaccine effectiveness better, inform strategies for healthcare worker (HCW) vaccination, and improve the understanding of the impact of COVID-19 on health programmes through the SLL programme. This sub-study focuses on the vaccination uptake, barriers and enablers among Ethiopian healthcare cadres.

Although the WHO declared an end to COVID-19 as a public health emergency ‘with great hope’, as of 05 May 2023, it also stressed that it does not mean that the disease is no longer a global threat.^7^ It has been reported that COVID-19 has caused severe disruption of essential health services.^8^ Identifying enablers and enhancers of COVID-19 vaccination among HCWs guarantees the protection of HCWs and reduces the impact of the pandemic on healthcare provision and the safety of patients. A healthcare workforce with a positive attitude towards vaccination would encourage patients and the community to get vaccinated through health promotion. Besides, the information generated around barriers and enablers of COVID-19 vaccination can be leveraged to reduce the impact of any similar pandemic in the future.

Ethiopia launched the first round of COVID-19 vaccination in March 2021, prioritising HCWs. A study conducted in Addis Ababa from 01 to 10 March 2021, just before the vaccine’s introduction, revealed that nearly two-thirds of HCWs (60.3%) were hesitant to take the COVID-19 vaccine. Coronavirus disease 2019 vaccine hesitancy increases among younger HCWs other than medical doctors and nurses, among those who lack belief in the benefits of the vaccine, lack trust in the government, lack of trusting science to produce safe and effective vaccines, and have a concern about the vaccine’s safety.^9^ The study from Gondar, Northwest Ethiopia, revealed an acceptance rate of 66.2% (33.8% hesitancy) among HCWs.^10^ In the same region, around the same duration (May 2021 – June 2021), 45.9% (*n* = 192) of vaccine hesitancy was reported among health professionals.^11^ A study conducted from 01 March 2021 to 30 March 2021, on 420 medical and health science students attending Wolkite University, South Ethiopia,^12^ recorded almost the same hesitancy level. The reported COVID-19 vaccine hesitancy was 41.2% (95% confidence interval [CI]: 35.2% – 50.4%).

The reported factors associated with vaccine hesitancyw include younger age, being a healthcare worker other than medical doctors, a lack of belief or trust in vaccine safety and efficacy, and low-risk self-perception.^9,11^

These studies in Ethiopia were conducted before or immediately after the launch of the first round of vaccination. This study was carried out while the fourth round of vaccination was in progress, intending to identify the barriers and enhancers to COVID-19 vaccination among HCWs in Ethiopia. We measured both the vaccine uptake and reasons why HCWs become hesitant to use the available opportunity for vaccination. We also looked into the proportion of HCWs willing to recommend COVID-19 vaccination to eligible individuals in their community.

## Research methods and design

### Study design

This was a multicentre cross-sectional study conducted from May 2023 to July 2023 in Addis Ababa and Adama, Ethiopia, known to be epicentres of SARS-CoV-2 transmission in the country.

### Study setting

The study included 30 randomly selected health facilities – hospitals and health centres.

### Study population and sampling strategy

The target population was HCWs, including physicians, nurses, lab technicians, pharmacists, other health professionals (radiographers, anaesthetists) and community health workers.

A minimum sample size of 422 was calculated, aiming for 80% power and a 95% CI. The sample was increased to 600 to improve precision. Participants were proportionally allocated across Addis Ababa (*n* = 400) and Adama (*n* = 200).

A two-stage random sampling approach was used. Initially, health facilities were randomly selected. Then, HCWs from the facility lists served as the sampling frame for the second-stage random selection.

### Data collection

Data were collected during a single visit. After obtaining written informed consent, research assistants administered a structured questionnaire to assess:

- Demographics (age, gender, profession, facility type, location).
- Beliefs and perceptions about vaccine efficacy (COVID-19 vs. other diseases).
- Vaccination details (doses received, preferred vaccination sites).
- Likelihood of recommending vaccination.
- Confidence in addressing patient queries.
- Vaccination barriers and reasons for hesitancy and refusal.
- Trust in information sources on COVID-19 and vaccines.

The questionnaire was pre-tested and translated into local languages. Data were collected using mobile devices and entered directly into REDCap software.

### Data analysis

Data entry was performed in real-time for accuracy. Data cleaning and quality control procedures were implemented. STATA software (version 14) was used for analysis. Proportions of uptake, barriers and enhancers of vaccination were calculated, and Chi-square tests were used to assess differences in proportions. Multivariable modified Poisson regression analysis identified factors associated with vaccine acceptance (*p* < 0.05). Adjusted prevalence ratios (aPR) with 95% CI were reported. The reasons for hesitancy were summarised to inform communication strategies.

### Ethical considerations

Ethical clearance to conduct this study was obtained from the Ethiopian Public Health Institute Institutional Review Board (No. EPHI-IRB-486-2022). Participants were informed about the study’s aims and their right to withdraw at any time.

## Results

### Sociodemographic characteristics

A total of 600 HCWs participated in the study with a response rate of 100%. Over half were females (*n* = 342/600, 57.0%), and the median age interquartile range (IQR) of 30 (27–34) years. Most HCWs were recruited from hospitals (*n* = 396/600, 66.0%). About 67.2% were working in publicly owned health facilities, as detailed in Table 1.

**TABLE 1:** Characteristics of healthcare workers from Addis Ababa and Adama, Ethiopia, (*N* = 600) participating in this study from May 2023 to July 2023.

Variables	Frequency (*n*)	Percentage	95% CI for the percentage
**Sex**			
Male	258	43.0	39.1–47.0
Female	342	57.0	52.9–60.9
**Age (years)**			
20–24	42	7.0	5.2–9.3
25–29	256	42.7	38.8–46.7
30–34	175	29.1	25.7–32.9
35–39	88	14.7	12.0–17.7
40 >	39	6.5	4.8–8.8
**Profession type**			
Physician	180	30.0	26.5–33.8
Nursing and midwifery	120	20.0	16.9–23.4
Pharmacy	60	10.0	7.8–12.7
Laboratory	120	20.0	16.9–23.4
Community health workers	60	10.0	7.8–12.7
Others†	60	10.0	7.8–12.7
**Facility ownership**			
Public	403	67.2	63.3–70.8
Private	174	29.0	25.5–32.7
Private for not profit	23	3.8	2.6–5.7
**Facility type**			
Hospital	396	66.0	62.1–69.9
Health centre	204	34.0	30.3–37.9

CI, confidence interval.

† , Radiographers, anaesthetists, and so on.

### Vaccination status of healthcare workers

**FIGURE 1:**
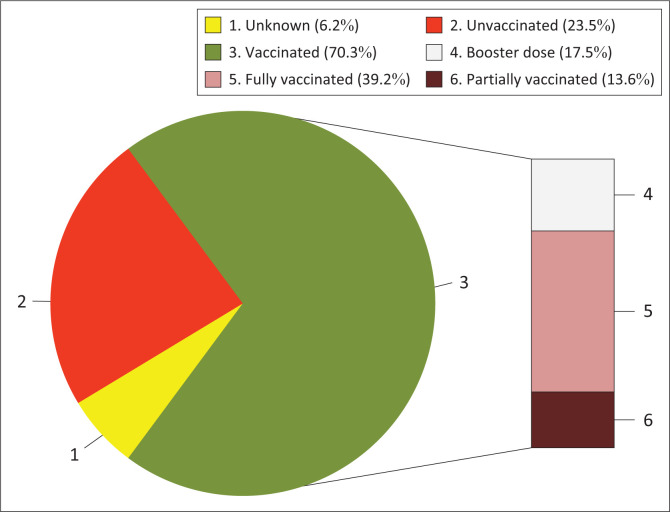
Vaccination status among healthcare workers from Addis Ababa and Adama, Ethiopia, (*N* = 600) participating in this study from May 2023 to July 2023.

The majority of HCWs 422 (70.3%, 95% CI: 66.5% – 73.9%) were either fully or partially vaccinated or have taken a booster dose. By vaccination status, 105 out of 600 (17.5%, 95% CI: 14.7% – 20.8%) HCWs had a booster dose, 235 out of 600 (39.2%, 95% CI: 35.3% – 43.1%) were fully vaccinated (one dose of a single-dose vaccine or two doses of ‘two-dose vaccine’), and 82 out of 600 (13.6%, 95% CI: 11.1% – 16.7%) were partially vaccinated (only one dose of ‘two-dose vaccine’) (Figure 1).

Participants were asked their reasons for not being fully vaccinated; these include all who were not fully vaccinated or received a booster dose (i.e. unknown – 37, unvaccinated – 141, partially vaccinated – 82), which gives a total of 260 HCWs. The majority of participants 135 (51.9%) disclosed that they were concerned about the safety of the vaccines, including side effects. Besides, 28 (10.8%) HCWs waiting to see how the vaccine affects other people that they know while 31 (11.9%) stated that they do not have time to get vaccinated (Table 2).

**TABLE 2:** Vaccination-related characteristics of healthcare workers from Addis Ababa and Adama, Ethiopia, May 2023 to July 2023.

Variables	Frequency (*n*)	Percentage
**Reasons for not getting vaccinated**		
I do not have transportation or I am unable to get to the vaccination site	4	1.5
Waiting for the window of eligibility to open	6	2.3
I do not want to miss work	8	3.1
I am concerned about the cost of vaccination	10	3.9
I do not know where to go to get a vaccine	13	5.0
I am waiting to see how the vaccine affects other people that I know	28	10.8
I do not have time to get vaccinated	31	11.9
I am concerned about the safety of the vaccine, including side effects	135	51.9
I do not wish to respond	33	12.7
Other reason, please specify	18	6.9
**Factors that help decision to get vaccinated**		
Closer vaccine administration (distance)	19	7.3
Better resources for appointment scheduling	20	7.7
Nothing, I will not get vaccinated	49	18.9
Full approval of vaccine from regulatory authorities	71	27.3
More information on vaccine safety and efficacy	138	53.1
Other	15	5.8
**Recommend vaccine to patients and community**		
Definitely recommend	286	47.7
Probably recommend	188	31.3
Probably not recommend	36	6.0
Definitely not recommend	35	5.8
I do not know	29	4.8
I do not wish to respond	26	4.3
**Recommended booster to patients and community**		
Recommend the booster vaccine doses recommended by the MoH in my country	222	37.0
Recommend booster vaccine doses recommended by the WHO	203	33.8
Recommend some booster vaccine	16	2.7
Vaccine doses are not available	11	1.8
Not recommend booster vaccine doses	51	8.5
I am not sure	97	16.2
**Reasons for not wanting to receive an approved COVID-19 vaccine and/or booster**		
I want a different vaccine than the one(s) available in my country/locality now	2	1.1
The vaccine available in my country will not protect me	3	1.7
People in my community are fearful about being near people who have been vaccinated	3	1.7
Someone in my family or community does not want me to get the vaccine	3	1.7
Vaccines can give you the disease they are designed to protect you against	5	2.8
Vaccines are against my religious beliefs	8	4.5
Not applicable	8	4.5
I am confident there will be other effective treatments soon	9	5.1
I already had COVID-19 and am not worried about being infected again	9	5.1
Other	11	6.2
Other concerns about conspiracies they might have heard about including micro-chips	12	6.7
I do not feel I am at risk of catching the virus	15	8.4
I do not feel I am at risk of getting very sick or dying from the virus	20	11.2
I do not yet know enough about the vaccine to decide	37	20.8
I feel the development and/or authorisation of the vaccine was rushed, and it may not be thoroughly tested	45	25.3
I am concerned about serious side effects, like blood clots, neurological disorders	56	31.5

COVID-19, coronavirus disease 2019; MoH, Ministry of Health, WHO, World Health Organization.

When participants were asked about factors that help their decision to get vaccinated, 138 (53.1%) stated that more information on vaccine safety and efficacy, 71 (27.3%) full approval of vaccine from regulatory authorities, while 49 (18.9%) stated that they will not get vaccinated, or nothing will help in their decision of being vaccinated.

Only 286 out of 600 (47.7%) HCWs would definitely recommend COVID-19 vaccination to the community or patients. While 222 (37.0%) HCWs would recommend booster vaccine doses, 51 (8.5%) would not, and 97 (16.2%) HCWs were not sure (Table 2).

**FIGURE 2:**
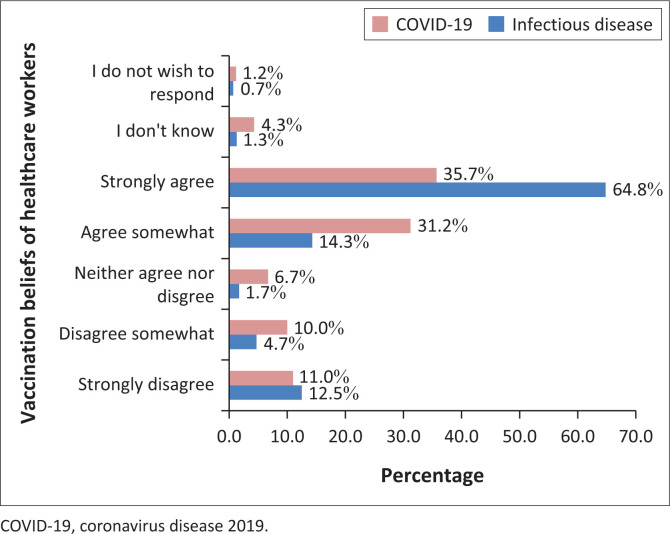
Healthcare workers belief about being vaccinated and prevention of getting sick or dying from infectious diseases and coronavirus disease 2019 from Addis Ababa and Adama, Ethiopia, May 2023 to July 2023.

Figure 2 displays HCWs’ belief about being vaccinated and prevention of getting sick or dying from infectious diseases in general and COVID-19 in particular. While about 65% (*n* = 389/600) of the HCWs strongly agree that being vaccinated has a protective role against infectious diseases only 35.7% (*n* = 214/600) strongly believe that being vaccinated for COVID-19 prevents getting sick or dying from it. On the other hand, a large proportion (11% for COVID-19 and 12.5% for infectious diseases) strongly disagree about the protective role of vaccination (Figure 2).

**FIGURE 3:**
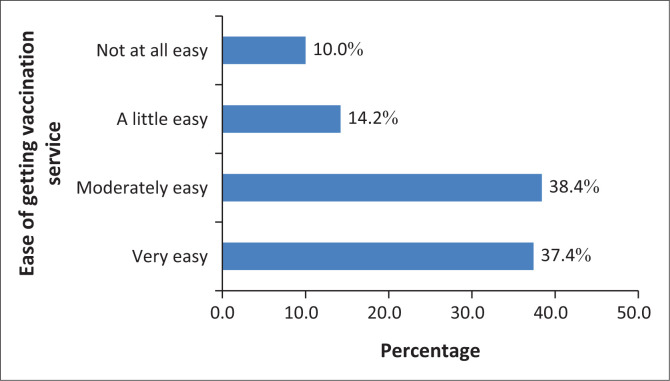
Ease of getting vaccination service among healthcare workers from Addis Ababa and Adama, Ethiopia that participated in the study conducted between May 2023 and July 2023.

Healthcare workers were also asked how easy it was to get vaccination services. Almost a quarter of them found it either a little easy or not at all easy (Figure 3).

**FIGURE 4:**
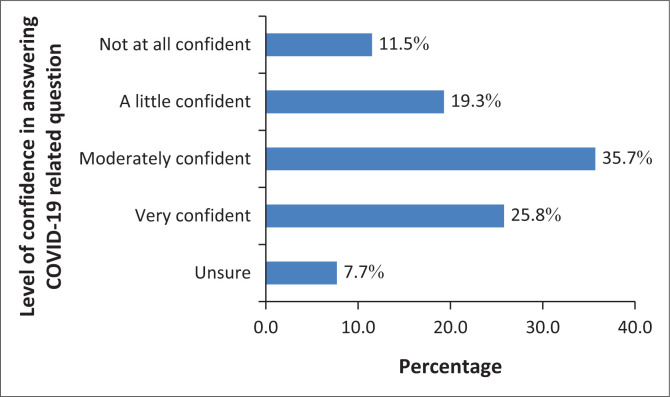
Confidence of healthcare workers in answering patient questions about locally available coronavirus disease 2019 vaccines from Addis Ababa and Adama, Ethiopia, May 2023 to July 2023.

Regarding confidence to answer questions about COVID-19, only 25.8% (*n* = 155/600) HCWs were very confident. About 31% of HCWs had either none or little confident while close to 8% were unsure of answering patient questions about locally available COVID-19 vaccinations as depicted in Figure 4.

### Factors associated with acceptance of COVID-19 vaccines among healthcare workers

Chi-square test analysis after excluding those HCWs with unknown vaccination status (21 who did not want to respond plus 16 who responded ‘Do not know’) revealed that factors like sex, age, profession type, facility ownership, facility type, recommendation of vaccine to patients, and confidence in answering questions were significantly associated(α = 0.05) with vaccination status of healthcare workers (Table 3).

**TABLE 3:** Chi-square analysis of sociodemographic characteristics and vaccination status of health care workers from Addis Ababa and Adama, Ethiopia (*n* = 563), May 2023 to July 2023.

Variables	Total		COVID-19 vaccination status						*P*
			Unvaccinated		Partially vaccinated		Fully vaccinated		
	*n*	%	*n*	%	*n*	%	*n*	%	
**Sex**	-	-	-	-	-	-	-	-	0.001
Male	248	44.1	46	32.6	32	39.00	170	50.00	-
Female	315	55.9	95	67.4	50	61.00	170	50.00	-
**Age (years)**	-	-	-	-	-	-	-	-	< 0.001
20–24	38	6.8	26	18.4	3	3.66	9	2.65	-
25–29	240	42.6	67	47.5	43	52.44	130	38.24	-
30–34	164	29.1	28	19.9	28	34.15	108	31.76	-
35–39	83	14.7	12	8.5	7	8.54	64	18.82	-
40 >	38	6.8	8	5.7	1	1.22	29	8.53	-
**Profession type**	-	-	-	-	-	-	-	-	0.001
Physician	177	31.4	30	21.3	28	34.15	119	35.00	-
Nursing and midwifery	111	19.7	24	17.0	22	26.83	65	19.12	-
Pharmacy	55	9.8	19	13.5	4	4.88	32	9.41	-
Laboratory	110	19.6	34	24.1	16	19.51	60	17.65	-
Community health workers	59	10.5	12	8.5	9	10.98	38	11.18	-
Others†	51	9.0	22	15.6	3	3.66	26	7.65	-
**Facility ownership**	-	-	-	-	-	-	-	-	0.005
Public	382	67.9	82	58.1	58	70.73	242	71.18	-
Private	160	28.4	55	39.0	20	24.39	85	25.00	-
Private for not profit	21	3.7	4	2.8	4	4.88	13	3.82	-
**Facility type**	-	-	-	-	-	-	-	-	< 0.001
Hospital	374	66.4	114	80.9	49	59.76	211	62.06	-
Health centre	189	33.6	27	19.1	33	40.24	129	37.94	-
**Recommend vaccine to patients and community**	-	-	-	-	-	-	-	-	< 0.001
Definitely recommend	275	48.9	38	26.9	41	50.00	196	57.65	-
Probably recommend	176	31.3	52	36.9	24	29.27	100	29.41	-
Probably not recommend	34	6.0	13	9.2	7	8.54	14	4.12	-
Definitely not recommend	28	5.0	15	10.6	2	2.44	11	3.24	-
I do not know	24	4.3	10	7.1	3	3.66	11	3.24	-
I do not wish to respond	26	4.6	13	9.2	5	6.10	8	2.35	-
**Recommended booster to patient and community**	-	-	-	-	-	-	-	-	< 0.001
Recommend the booster vaccine doses recommended by the MoH in my country	211	37.5	28	19.9	32	39.02	151	44.41	-
Recommend booster vaccine doses recommended by the WHO	190	33.8	42	29.8	31	37.80	117	34.41	-
Recommend some booster vaccine	16	2.8	1	0.7	1	1.22	14	4.12	-
Vaccine doses are not available	10	1.8	5	3.6	1	1.22	4	1.18	-
Not recommend booster vaccine doses	51	9.1	29	20.6	5	6.10	17	5.00	-
I am not sure	85	15.1	36	25.5	12	14.63	37	10.88	-
**Confidence in answering questions**	-	-	-	-	-	-	-	-	< 0.001
Not at all confident	61	10.8	24	17.0	9	10.98	28	8.24	-
A little confident	107	19.0	30	21.3	15	18.29	62	18.24	-
Moderately confident	203	36.1	41	29.1	28	34.15	134	39.41	-
Very confident	149	26.5	23	16.3	22	26.83	104	30.59	-
Unsure	43	7.6	23	16.3	8	9.76	12	3.53	-

MoH, Ministry of Health; WHO, World Health Organization.

† , Radiographers, anaesthetists, and so on.

The results from the final multivariable modified Poisson regression analysis revealed that the covariates: age, profession type, facility type, vaccine recommendation, a booster dose recommendation were associated with the acceptance of COVID-19 vaccine among the HCWs. The rate of having vaccination is two times higher for HCWs with the ages of 25–29 years, 30–34 years, 35–39 years, and 40 years and above had prevalence ratio (PR) 2.084 (CI: 1.320–3.288, *p*-value = 0.002), 2.337 (CI: 1.480–3.691, *p*-value = 0.0001), 2.256 (CI: 1.424–3.573, *p*-value = 0.001) and 2.163 (CI: 1.346–3.477, *p*-value = 0.001) times higher rate of having COVID-19 vaccination as compared to HCWs with age 20–24 years.

Physicians had a better vaccination acceptance rate as compared to pharmacy (PR = 0.787, CI: 0.650–0.952, *p*-value = 0.014), laboratory (PR = 0.848, CI: 0.738–0.974, *p*-value = 0.019) and other health workers (PR = 0.775, CI: 0.618–0.970, *p*-value = 0.026). Healthcare workers working in health centres were 1.201 (CI: 1.076–1.341, *p*-value = 0.001) times more likely to be vaccinated than HCWs working in hospitals (Table 3). Healthcare workers who would not recommend booster vaccine doses to patients and the community were 41% less likely to get vaccinated (PR = 0.590, CI: 0.411–0.845, *p*-value = 0.004). Besides, those who were unsure whether or not they recommended a booster dose vaccine were less likely to be vaccinated with a borderline significance (PR = 0.720, CI: 0.506–1.024, *p*-value = 0.067) (Table 4).

**TABLE 4:** Modified Poisson regression analysis of sociodemographic characteristics and vaccination status of healthcare workers from Addis Ababa and Adama, Ethiopia (*n* = 563), May 2023 to July 2023.

Variables	CPR	95% CI CPR	APR	95% CI APR	*P*
**Sex**					
Female	1	1	1	1	-
Male	1.166	1.062–1.281	1.081	0.981–1.191	0.114
**Age (years)**					
20–24	1	1	1	1	-
25–29	2.283	1.420–3.671	2.084	1.320–3.288	0.002
30–34	2.626	1.635–4.217	2.337	1.480–3.691	0.0001
35–39	2.709	1.682–4.363	2.256	1.424–3.573	0.001
40 and above	2.500	1.522–4.107	2.163	1.346–3.477	0.001
**Profession type**					
Physician	1	1	1	1	-
Nursing and midwifery	0.944	0.838–1.062	0.968	0.850–1.101	0.618
Pharmacy	0.788	0.643–0.965	0.787	0.650–0.952	0.014
Laboratory	0.832	0.723–0.959	0.848	0.738–0.974	0.019
Community health workers	0.959	0.830–1.109	0.871	0.735–1.033	0.112
Others	0.685	0.534–0.878	0.775	0.618–0.970	0.026
**Facility ownership**					
Public	1	1	1	1	-
Private	0.835	0.738–0.946	0.954	0.854–1.065	0.403
Private for not profit	1.031	0.832–1.277	1.092	0.862–1.384	0.466
**Facility type**					
Hospital	1	1	1	1	-
Health centre	1.233	1.128–1.348	1.201	1.076–1.341	0.001
**Recommend vaccine to patients and community**					
Definitely recommend	1	1	1	1	-
Probably recommend	0.818	0.735–0.910	0.910	0.821–1.009	0.073
Probably not recommend	0.717	0.548–0.938	0.972	0.759–1.245	0.825
Definitely not recommend	0.539	0.361–0.805	0.787	0.522–1.186	0.252
I do not know	0.677	0.481–0.953	0.943	0.676–1.317	0.732
I do not wish to respond	0.580	0.394–0.855	0.780	0.527–1.154	0.214
**Recommended booster to patient and community**					
Recommend booster vaccine doses recommended by MoH	1	1	1	1	-
Recommend booster vaccine doses recommended by WHO	0.898	0.819–0.985	0.931	0.854–1.015	0.103
Recommend some booster vaccine scheduled and not others	1.081	0.942–1.240	1.080	0.941–1.239	0.276
Booster vaccine doses are not available in my country	0.577	0.309–1.074	0.769	0.470–1.259	0.297
Would not recommend booster vaccine doses	0.497	0.361–0.685	0.590	0.411–0.845	0.004
I am not sure	0.665	0.550–0.804	0.840	0.698–1.009	0.063
**Confidence in answering questions**					
Not at all confident	1	1	1	1	-
A little confident	1.186	0.939–1.499	0.991	0.807–1.216	0.929
Moderately confident	1.316	1.062–1.629	1.0412	0.861–1.258	0.680
Very confident	1.394	1.126–1.726	1.003	0.824–1.220	0.977
Unsure	0.767	0.525–1.120	0.720	0.506–1.024	0.067

APR, adjusted prevalence ratio; CPR, crude prevalence ratio; CI, confidence interval; MoH, Ministry of Health; WHO, World Health Organization.

## Discussion

Healthcare workers are significantly at a higher risk of infection and transmission because of their frequent and close contact with highly contagious COVID-19 patients.^13^ Vaccination is the best and least expensive protection against infectious diseases, including COVID-19, and is one of the most significant advancements in enhancing public health.^14^ The principal findings of this study indicated that one-third of the HCWs in the study area did not receive COVID-19 vaccination. As HCWs are front-line responders and trusted individuals by the general population, their hesitancy has serious public health implications. Notably, response to public health emergencies requires a multidisciplinary team, effective collaboration and deployment of an optimal health workforce. The infection of this health workforce, coupled with the already very low HCWs-to-patient ratios, may complicate the response efforts. Moreover, as the HCWs are considered a reliable source of information by the general public, their hesitancy might directly impact the vaccine hesitancy of the general population – jeopardising the pandemic response efforts.

The 70.3% vaccination status reported in this study is consistent with findings of studies from other sub-Saharan African countries such as Ghana (70%)^15^ and Ethiopia (74.64%)^16^ as well as a global meta-analysis that reported a 72.1% average for African countries included.^17^ However, the result of this study was lower than result of findings from Chicago (85%),^18^ Canada (80.9%),^19^ and China (76.98%).^20^ On the other hand, an extremely low COVID-19 vaccine acceptance rate was reported from the Democratic Republic of Congo (27.7%),^21^ the United States (US) (36%),^22^ north-eastern Ethiopia (52.3%)^23^ Iraq, (61.7%),^24^ the Kingdom of Saudi Arabia (64.9%),^25^ North Central Ethiopia (65%).^26^ The possible explanations could be methodology, sample size, and varying concerns of HCWs about vaccine safety, efficacy, and speed or rash in vaccine development. For example, the study from DRC was carried out from March 2020 to 30 April 2020, before the introduction of the COVID-19 vaccine^21^; thus, it is more likely for HCWs to be hesitant as they may need to be well-informed about the vaccines. In support of this explanation, the study from the US collected data between 07 October 2020 and 09 November 2020, and more than half of the HCWs (56%) were not sure or would wait to review more data on COVID-19 vaccines to be willing to take the vaccine as soon as it became available.^22^

In consistency with many other studies, the majority (51.9%) disclosed that the main reason for not getting vaccinated is concern about the safety of the vaccine, including its side effects.^25,27,28^ These findings imply that for any emerging pandemics, developing trust of the HCWs about the vaccine development process and awareness about the risk of potential side effects versus saving professionals from danger is vital. The finding in this study also supported this explanation; about 53.1% of HCWs stated more information on vaccine safety and efficacy could help them decide to get vaccinated and close to a quarter of them (27.3%) suggested full approval of vaccine from regulatory authorities would help them decide to get vaccinated. Of interest and high concern, 18.9% HCWs stated nothing will help to change their decision of not to be vaccinated. This finding was high when we compare with the findings from United States (8%)^22^ and Italian medical students (6.7%).^29^ This needs critical attention by the Ministry of Health to inform future pandemic preparedness package as vaccines are the mainstay in the prevention and control.

Healthcare workers were supposed to influence the community regarding health interventions. They are expected to be the first line of response to promote and encourage the community to practice interventions and guidelines set by the health ministry. However, in this study only 47.7% of the HCWs said they would definitely recommend COVID-19 vaccination to the community or patients and only a quarter of the respondents were confident while 11.5% were not at all confident to answer questions related to COVID-19. The evidence by Berry M, et al. supports this finding.^30^ This study also revealed a concerning finding: just 35.7% of HCWs believed that the COVID-19 immunisation prevented illness or death, whereas 64.8% believed that vaccination against other infectious diseases did the same. This is a major gap that has to be addressed both for the routine Expanded Programme on Immunization (EPI) and any future intervention because misconceptions against vaccinations could hamper interventions against any emerging infectious diseases.

Finally, the covariates age (being older than 25 years), profession type (being medical doctor), facility type (working in health centres), and vaccine recommendation have significant impact on the acceptance of COVID-19 vaccine. Those aged more than 25 years were more than twice more likely to be vaccinated. This may imply differences in risk perception and concurred with another study from Ethiopia,^31^ although a contradicting finding was also reported where younger doctors, in Italy both in age and experience were more confident in vaccines and recommended them more frequently.^32^ Possibly Ethiopia being among the African countries with young population, the demographic difference between the two populations might explain the observed variations. Supporting the likeliness of such explanation, what is indicated as young adults according to the Italian study is an average age of 38.8 ± 11.32 years, while the median and IQR of our study is 30 (27–34) years.

Medical doctors’ better acceptance of vaccine also concurred with results of Ulbrichtova, R et al. that physicians (odds ratio [OR] = 1.77; 95% CI: 1.13–2.78) have higher vaccination acceptance rates and lower hesitance to get vaccinated than non-physician HCWs.^33^ The reason why HCWs working in health centres than hospitals were more likely to be vaccinated could be associated with accessibility of vaccines as indicated in this study. This finding contradicts the observation of Tolossa et al. (2022) from Southwest Ethiopia where those working in health centre (adjusted odds ration [AOR] = 2.45, 95% CI: 1.01, 5.92, *p*-value = 0.045) were associated with unfavourable attitude towards COVID-19 vaccine.^34^

This study’s limitation is that we restricted our focus solely to HCWs, overlooking the broader spectrum of multisectoral involvement. Furthermore, because of a small sample size, generalisability of the findings may be limited. However, because a quite large proportion of HCWs (*n* = 135, 51.9%) reported concern about the safety of the vaccine, including side effects, which they accounted to the rush in vaccine production; 37 (20.8%) do not yet know enough about the vaccine to decide; 45 (25.3%) feel the development and/or authorisation of the vaccine was rushed, and it may not be thoroughly tested, and 56 (31.5%) were concerned about serious side effects, like blood clots, and neurological disorders.

## Conclusion

The findings in this study have implications to frame our interventions. It identifies concerns that must be addressed to boost HCWs’ confidence on intervention strategies and protect themselves as well as to communicate better to their patients and the community about new pandemics such as COVID-19. Moreover, efforts have to be made to improve the ease of vaccine accessibility to HCWs because almost a quarter of the respondents reported that vaccine accessibility was either little easy or not at all easy. A substantial proportion of the HCWs in Addis Ababa and Adama, Ethiopia were hesitant to receive COVID-19 vaccines. As hesitancy is associated with young age, profession, facility type, and a lack of confidence to recommend to others, there is a need for targeted awareness creation and sensitisation for an increased uptake of the vaccines. More specifically, capacitating HCWs about the vaccine production process, which leverages the existing knowledge, the regulatory process in pandemic situations and potential side effects as well as how they can be managed through continuous professional development (CPD) by professional associations, the Ministry of Health and all stakeholders can be potential strategies to increase acceptance. The findings can be leveraged to maximise the protection of vulnerable populations against COVID-19 and any future similar pandemics.

## References

[1] World Health Organization. Coronavirus disease (COVID-19) [homepage on the Internet]. [cited 2024 May 03]. Available from: https://www.who.int/emergencies/diseases/novel-coronavirus-2019

[2] World Health Organization. COVID-19 cases | WHO COVID-19 dashboard [homepage on the Internet]. WHO Data. [cited 2024 May 03]. Available from: https://data.who.int/dashboards/covid19/cases

[3] Liu Q, Qin C, Liu M, Liu J. Effectiveness and safety of SARS-CoV-2 vaccine in real-world studies: A systematic review and meta-analysis. Infect Dis Poverty. 2021;10(1):132. 10.1186/s40249-021-00915-3 34776011 PMC8590867

[4] Dye C. The benefits of large scale covid-19 vaccination. BMJ. 2022;377:o867. 10.1136/bmj.o867 35477535

[5] World Health Organization. Global COVID-19 vaccination strategy in a changing world: July 2022 update [homepage on the Internet]. 2022 [cited 2024 May 03]. Available from: https://www.who.int/publications/m/item/global-covid-19-vaccination-strategy-in-a-changing-world--july-2022-update

[6] Ackah BBB, Woo M, Stallwood L, et al. COVID-19 vaccine hesitancy in Africa: A scoping review. Glob Health Res Policy. 2022;7(1):21. 10.1186/s41256-022-00255-1 35850783 PMC9294808

[7] Unite Nations. WHO chief declares end to COVID-19 as a global health emergency | UN News [serial online]. UN News. 2023 [cited 2024 May 03]. Available from: https://news.un.org/en/story/2023/05/1136367?_gl=1*ukf4m3*_ga*MjAzMTk5MTMxMi4xNzE0NzI4OTI4*_ga_TK9BQL5X7Z*MTcxNDcyODkyNy4xLjAuMTcxNDcyOTE1MC4wLjAuMA

[8] Derseh A, Id M, Nega A, et al. Effect of the COVID-19 pandemic on health service utilization across regions of Ethiopia: An interrupted time series analysis of health information system data from 2019–2020. PLOS Glob Public Health. 2022;2(9):e0000843. 10.1371/journal.pgph.0000843 36962800 PMC10021875

[9] Mohammed R, Nguse TM, Habte BM, Fentie AM, Gebretekle GB. COVID-19 vaccine hesitancy among Ethiopian healthcare workers. PLoS One. 2021;16(12):e0261125. 10.1371/journal.pone.0261125 34919597 PMC8682893

[10] Demeke CA, Kifle ZD, Atsbeha BW, et al. COVID-19 vaccine hesitancy among health professionals in a tertiary care center at the University of Gondar Specialized Hospital, Ethiopia: A cross-sectional study. SAGE Open Med. 2022;10:20503121221076991. 10.1177/20503121221076991 35186292 PMC8855372

[11] Aemro A, Amare NS, Shetie B, Chekol B, Wassie M. Determinants of COVID-19 vaccine hesitancy among health care workers in Amhara region referral hospitals, Northwest Ethiopia: A cross-sectional study. Epidemiol Infect. 2021;149:e225. 10.1017/s0950268821002259 34645533 PMC8548232

[12] Mose A, Haile K, Timerga A. COVID-19 vaccine hesitancy among medical and health science students attending Wolkite University in Ethiopia. PLoS One. 2022;17(1):e0263081. 10.1371/journal.pone.0263081 35077504 PMC8789154

[13] Nguyen LH, Drew DA, Graham MS, et al. Risk of COVID-19 among front-line health-care workers and the general community: A prospective cohort study. Lancet Public Health. 2020;5(9):e475–e483. 10.1016/s2468-2667(20)30164-x 32745512 PMC7491202

[14] Giubilini A, Savulescu J. Vaccination, risks, and freedom: The seat belt analogy. Public Health Ethics. 2019;12(3):237–249. 10.1093/phe/phz014 32082418 PMC7020768

[15] Alhassan RK, Owusu-Agyei S, Ansah EK, Gyapong M. COVID-19 vaccine uptake among health care workers in Ghana: A case for targeted vaccine deployment campaigns in the global south. Hum Resour Health. 2021;19(1):136. 10.1186/s12960-021-00657-1 34742301 PMC8571849

[16] Figa Z, Temesgen T, Zemeskel AG, et al. Acceptance of COVID-19 vaccine among healthcare workers in Africa, systematic review and meta-analysis. Public Health Pract (Oxf). 2022;4:100343. 10.1016/j.puhip.2022.100343 36438628 PMC9681992

[17] Galanis P, Vraka I, Katsiroumpa A, et al. COVID-19 vaccine uptake among healthcare workers: A systematic review and meta-analysis. Vaccines (Basel). 2022;10(10):1637. 10.3390/vaccines10101637 36298502 PMC9610263

[18] Toth-Manikowski SM, Swirsky ES, Gandhi R, Piscitello G. COVID-19 vaccination hesitancy among health care workers, communication, and policy-making. Am J Infect Control. 2022;50(1):20–25. 10.1016/j.ajic.2021.10.004 34653527 PMC8511871

[19] Dzieciolowska S, Hamel D, Gadio S, et al. Covid-19 vaccine acceptance, hesitancy, and refusal among Canadian healthcare workers: A multicenter survey. Am J Infect Control. 2021;49(9):1152–1157. 10.1016/j.ajic.2021.04.079 33930516 PMC8079260

[20] Wang MW, Wen W, Wang N, et al. COVID-19 vaccination acceptance among healthcare workers and non-healthcare workers in China: A survey. Front Public Health. 2021;9:709056. 10.3389/fpubh.2021.709056 34409011 PMC8364953

[21] Kabamba Nzaji M, Kabamba Ngombe L, Ngoie Mwamba G, et al. Acceptability of vaccination against COVID-19 among healthcare workers in the Democratic Republic of the Congo. Pragmat Obs Res. 2020;11:103–109. 10.2147/POR.S271096 33154695 PMC7605960

[22] Shekhar R, Sheikh AB, Upadhyay S, et al. COVID-19 vaccine acceptance among health care workers in the United States. Vaccines (Basel). 2021;9(2):119. 10.3390/vaccines9020119 33546165 PMC7913135

[23] Adane M, Ademas A, Kloos H. Knowledge, attitudes, and perceptions of COVID-19 vaccine and refusal to receive COVID-19 vaccine among healthcare workers in northeastern Ethiopia. BMC Public Health. 2022;22(1):128. 10.1186/s12889-021-12362-8 35042476 PMC8765812

[24] Al-Metwali BZ, Al-Jumaili AA, Al-Alag ZA, Sorofman B. Exploring the acceptance of COVID-19 vaccine among healthcare workers and general population using health belief model. J Eval Clin Pract. 2021;27(5):1112–1122. 10.1111/jep.13581 33960582 PMC8242385

[25] Elharake JA, Galal B, Alqahtani SA, et al. COVID-19 vaccine acceptance among health care workers in the Kingdom of Saudi Arabia. Int J Infect Dis. 2021;109:286–293. 10.1016/j.ijid.2021.07.004 34242765 PMC8260488

[26] Kebede SD, Aytenew TM. Attitude, knowledge, and predictors of COVID-19 vaccine uptake among health care providers in South Gondar public hospitals, North Central Ethiopia: Multi-facility based study. Pan Afr Med J. 2022;41:194. 10.11604/pamj.2022.41.194.30868 35685100 PMC9146589

[27] Meyer MN, Gjorgjieva T, Rosica D. Trends in health care worker intentions to receive a COVID-19 vaccine and reasons for hesitancy. JAMA Netw Open. 2021;4(3):e215344. 10.1001/jamanetworkopen.2021.5344 33755164 PMC7988365

[28] Youssef D, Abou-Abbas L, Berry A, Youssef J, Hassan H. Determinants of acceptance of coronavirus disease-2019 (COVID-19) vaccine among Lebanese health care workers using health belief model. PLoS One. 2022;17(2):e0264128. 10.1371/journal.pone.0264128 35192664 PMC8863223

[29] Lo Moro G, Cugudda E, Bert F, Raco I, Siliquini R. Vaccine hesitancy and fear of COVID-19 among Italian medical students: A cross-sectional study. J Community Health. 2022;47(3):475–483. 10.1007/s10900-022-01074-8 35138490 PMC9160103

[30] Barry M, Temsah MH, Alhuzaimi A, et al. COVID-19 vaccine confidence and hesitancy among health care workers: A cross-sectional survey from a MERS-CoV experienced nation. PLoS One. 2021;16(11):e0244415. 10.1371/journal.pone.0244415 34843462 PMC8629228

[31] Getachew T, Lami M, Eyeberu A, et al. Acceptance of COVID-19 vaccine and associated factors among health care workers at public hospitals in Eastern Ethiopia using the health belief model. Front Public Health. 2022;10:957721. 10.3389/fpubh.2022.957721 36438218 PMC9683340

[32] Rapisarda V, Vella F, Ledda C, Barattucci M, Ramaci T. What prompts doctors to recommend COVID-19 vaccines: Is it a question of positive emotion? Vaccines (Basel). 2021;9(6):578. 10.3390/vaccines9060578 34205935 PMC8229710

[33] Ulbrichtova R, Svihrova V, Tatarkova M, Hudeckova H, Svihra J. Acceptance of COVID-19 vaccination among healthcare and non-healthcare workers of hospitals and outpatient clinics in the Northern Region of Slovakia. Int J Environ Res Public Health. 2021;18(23):12695. 10.3390/ijerph182312695 34886420 PMC8657382

[34] Tolossa T, Wakuma B, Turi E, et al. Attitude of health professionals towards COVID-19 vaccination and associated factors among health professionals, Western Ethiopia: A cross-sectional survey. PLoS One. 2022;17(3):e0265061. 10.1371/journal.pone.0265061 35263375 PMC8906598

